# Two new species of the *Stenochinus amplus* species-group from China (Coleoptera, Tenebrionidae, Stenochiini)

**DOI:** 10.3897/zookeys.416.7568

**Published:** 2014-06-17

**Authors:** Cai-Xia Yuan, Guo-Dong Ren

**Affiliations:** 1 College of Life Sciences, Hebei University, Baoding, 071002, P. R. China; 2 College of Life Sciences, Yan’an University, Yan’an 716000, P. R. China

**Keywords:** Tenebrionidae, Stenochiini, *Stenochinus*, new species, China

## Abstract

Two new species of the *Stenochinus amplus* species-group are described, *S. apiciconcavus* **sp. n.** (CHINA: Shaanxi) and *S. xinyicus* **sp. n.** (CHINA: Guangdong). Also, some new distribution data are provided for *S. cylindricus* (Gebien, 1914), and a key to the seven species of the *S. amplus* species-group from China is given.

## Introduction

The genus *Stenochinus* was established by Motschulsky (1860) for *Stenochinus reticulatus*. Contributions to the taxonomy of this genus have been made by several workers, such as Marseul (1876), Fairmaire (1896), Gebien (1911, 1914, 1925), Gravely (1915), Pic (1921, 1922, 1923, 1926, 1927, 1958), Kulzer (1954), Nakane (1956, 1985), Ardoin (1969), Kaszab (1978, 1980a, 1980b), Shibata (1980), Merkl (1992), Masumoto and Akita (2002), Ren and Yang (2004), Schawaller (2006), Löbl et al. (2008) and Masumoto et al. (2013), this group currently includes 43 species and subspecies, which are mainly distributed in the Oriental Region. The species of Taiwan and Japan were divided into three species-groups by Shibata (1980), one of them, the *Stenochinus amplus* species-group has so far 12 species (Shibata 1980, Masumoto and Akita 2002, Ren and Yang 2004, Schawaller 2006, Masumoto et al. 2013). In the present study, two new species of this group from China are described. Besides, a key to the Chinese species of the *Stenochinus amplus* species-group is provided, including new distribution data for *Stenochinus cylindricus* (Gebien, 1914).

## Material and methods

Specimens were examined under a Nikon (SMZ800) dissecting microscope. Measurements were taken and photographs captured using a Leica (M205 A) dissecting microscope. The habitus photos were taken with a Canon (EOS 5D mark II) camera.

The measurements were as follows: body length: length of the body from the anterior edge of the clypeus to elytral apex with the head in its natural position; body width: length of the maximal elytral width; pronotal length: length of the pronotum along the midline; elytral length: length of the elytra from the base of the scutellum to the elytral apex along the suture. All measurements are given in millimeters.

The following codens of the collections are used:

MHBU Museum of Hebei University, Baoding, China;

SYSU Research Institute of Entomology, Sun Yat-Sen University, Guangzhou, China;

IZCAS Institute of Zoology, Chinese Academy of Sciences, Beijing, China.

## Taxonomy

### *Stenochinus amplus* species-group

**Diagnosis.** Body covered with distinct scale-like hairs, mandibles subtruncate, apical projections of pronotum not or hardly reaching anterior margins of eyes, tibiae with rows of suberect hairs along inner margins, male genitalia widened basally.

**Distribution.** China (including Taiwan), Japan, Vietnam, India, Nepal, Sri Lanka.

**Key to the species of *Stenochinus amplus* species-group in China**

**Table:** 

1	Elytra distinctly convex, oval. Brachypterous species	2
−	Elytra weakly convex, subcylindrical (Figs 17–20). Winged species	4
2	Anterior margin of pronotum distinctly emarginate	3
−	Anterior margin of pronotum without emargination	*Stenochinus mysticus* Masumoto, Akita & Lee
3	Body slender, gradually widened in basal one-fourth part of elytra	*Stenochinus akiyamai* Masumoto, Akita & Lee
−	Body stouter, subparallel-sided in basal one-fourth part of elytra	*Stenochinus amplus* (Gebien)
4	Pronotum moderately projecting forward (Figs 1–3, 5)	5
−	Pronotum strongly projecting forward (Figs 4, 6)	*Stenochinus cylindricus* (Gebien)
5	Anterior margin of pronotum without emargination (Figs 2–4). Scutellum subquadrate	6
−	Anterior margin of pronotum distinctly emarginate (Fig. 1). Scutellum subrectangular	*Stenochinus apiciconcavus* sp. n.
6	Pronotum weakly reflexed at anterior margin, scale-like hairs on pronotum longer and wider than those on elytra	*Stenochinus xinyicus* sp. n.
−	Pronotum not reflexed at anterior margin, scale-like hairs on pronotum nearly same as on elytra	*Stenochinus leprosus* (Ren & Yang)

#### Stenochinus apiciconcavus sp. n.

Taxon classificationAnimaliaColeopteraTenebrionidae

http://zoobank.org/3BD90C95-FA8E-4919-AF90-196DA9F0CF1E

**Figures 1−6.**
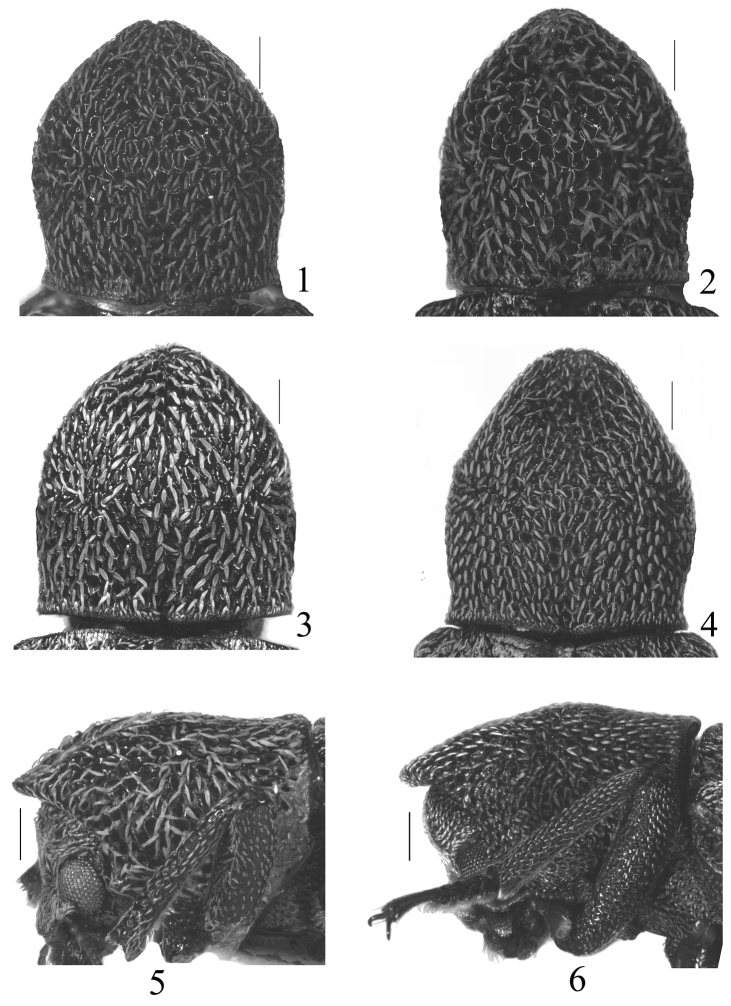
Pronotum. 1 *Stenochinus apiciconcavus* sp. n. **2−3, 5** *Stenochinus xinyicus* sp. n. (**2, 5** ♂ **3** ♀) **4, 6** *Stenochinus cylindricus*. Scale bars = 0.5 mm.

**Figures 7−16.**
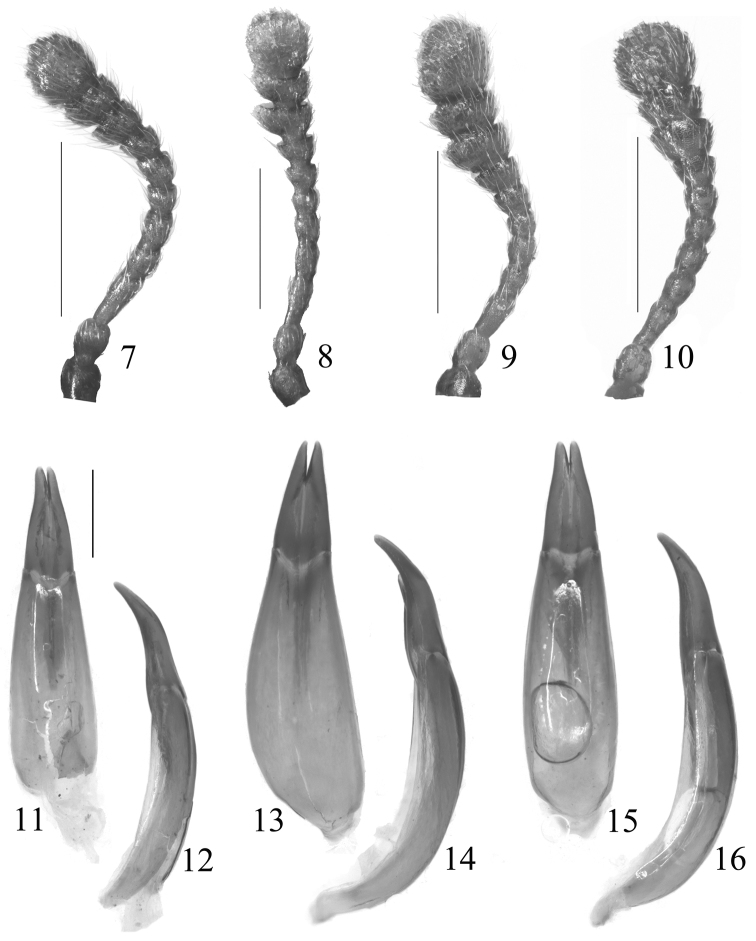
**7−10** Antennae **11−16** Male genitalia in dorsal view and lateral view **7, 11−12** *Stenochinus apiciconcavus* sp. n. **8−9, 13−14** *Stenochinus xinyicus* sp. n. (**8** ♂ **9** ♀) **10, 15−16** *Stenochinus cylindricus*. Scale bars = 0.5 mm

**Figures 17−20.**
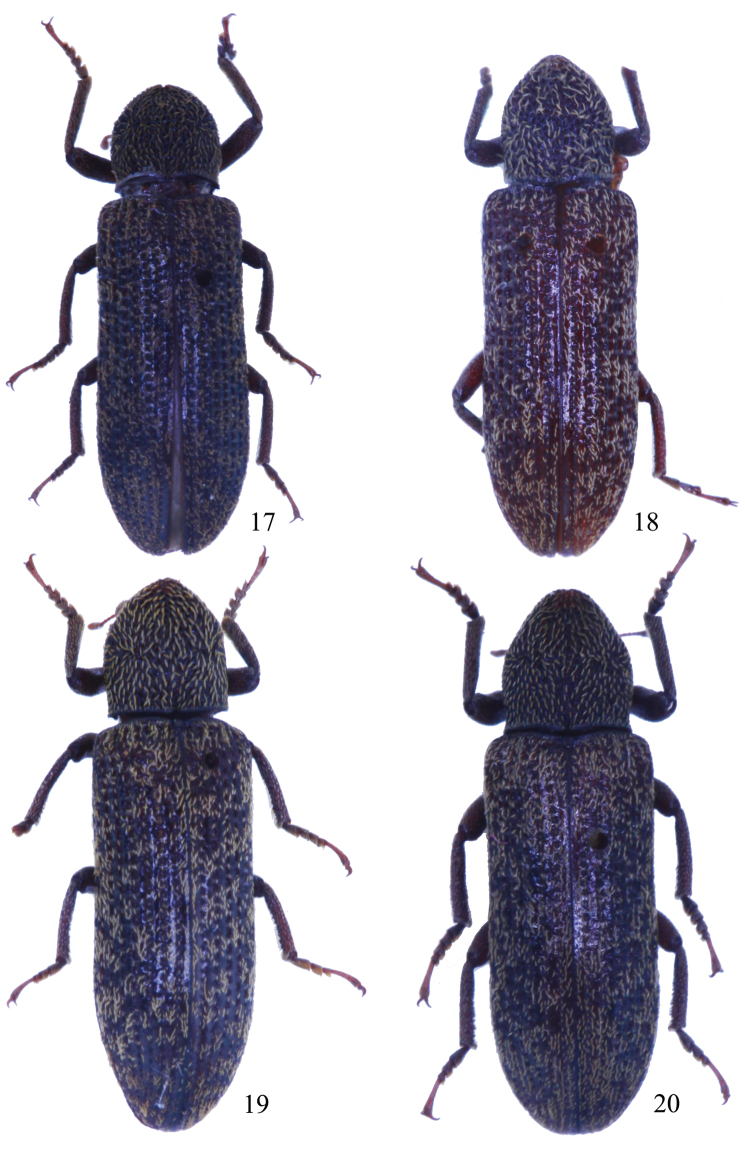
Habitus. **17** *Stenochinus apiciconcavus* sp. n. ♂ **18−19** *Stenochinus xinyicus* sp. n. (**18** ♂ **19** ♀) **20** *Stenochinus cylindricus* ♂.

Figs 1 , 7 , 11–12 , 17

##### Type material.

Holotype: ♂, China, Shaanxi, Nanzheng county, Beiba town, 20.vi.2005, Yi-Bin Ba leg. (MHBU).

##### Diagnosis.

This new species is similar to *Stenochinus cylindricus* (Gebien, 1914), but can be distinguished from the latter by the pronotum moderately projecting anteriorly, with emargination in middle of anterior margin; scutellum subrectangular; maxillary palpomere IV strongly expanded (in *Stenochinus cylindricus*, pronotum strongly projecting anteriorly, without emargination at anterior margin (Figs 4, 6); scutellum subquadrate; maxillary palpomere IV moderately expanded). It also resembles *Stenochinus wakoi* Masumoto & Akita, 2002, but differs in the following characters: antennomeres VIII–XI wider than long; pronotum with emargination in middle of anterior margin; scutellum subrectangular, glabrous (in *Stenochinus wakoi*, antennomeres VI–XI wider than long; pronotum without emargination at anterior margin; scutellum subquadrate, covered with scale-like hairs on both sides).

##### Etymology.

The specific name is derived from the Latin *apici* [apex] + *concavus*[concave], a reference to the pronotum with an emargination in middle of anterior margin.

##### Description.

Male (Fig. 17). Body length 10.3 mm, elongate, subcylindrical. Head, elytra and legs dark reddish brown, antennae reddish brown, pronotum dark brown. Scale-like hairs on the surface pale golden. Head transversely subelliptical, surface densely punctate; clypeus truncate at anterior margin, clypeogenal suture grooved, frontoclypeal suture invisible; genae weakly raised, with rounded outer margins; frons wide, distance between eyes 2.56 times as wide as transverse diameter of an eye in dorsal view. Eyes medium-sized, weakly protruding, each side with a groove along inner and posterior margins. Antennae (Fig. 7) clavate, antennomeres VIII–XI each wider than long, antennomeres XI ovate, ratio of the length of antennomeres II–XI as 0.12 : 0.18 : 0.09 : 0.09 : 0.07 : 0.08 : 0.07 : 0.10 : 0.08 : 0.22. Maxillary palpomere IV strongly expanded. Pronotum (Fig. 1) 1.13 times as long as wide, widest in middle; anterior margin with a distinct emargination in middle; posterior margin weakly bisinuate, with deep emargination in middle; both sides steeply inclined downward, lateral margins sinuate before posterior angles; anterior angles acute and directed anteriad, posterior angles acute and directed postero-laterad; disc subelliptically projecting in anterior parts, this projecting area distinctly impressed, surface roughly and deeply punctuate, punctures often fused with one anonther. Scutellum subrectangular, glabrous. Elytra 2.5 times as long as wide, widest at apical 1/3, 2.76 times as long as and 1.25 times as wide as pronotum; dorsum convex but flattened in lateral view; disc with rows of subquadrate punctures, which are larger and deeper anteriorly, each puncture with a granule on each lateral margin; intervals somewhat transversely wrinkled, weakly ridged in lateral parts, scale-like hairs on the intervals shorter than those on pronotum. Ventral side covered with dense punctures and scale-like hairs, which distinctly shorter than those on pronotum. Legs relatively short, ratio of the lengths of metatarsomeres I–IV as 0.45 : 0.24 : 0.17 : 0.69. Male genitalia (Figs 11–12) curved in middle in lateral view, 1.94 mm long, 0.43 mm wide; apicale 0.71 mm long, weakly curved in lateral view.

Female: Unknown.

#### Stenochinus xinyicus sp. n.

Taxon classificationAnimaliaColeopteraTenebrionidae

http://zoobank.org/D193ED7A-DE68-42F1-9BDE-F748463B88A3

Figs 2–3 , 5 , 8–9 , 13–14 , 18–19

##### Type material.

Holotype: ♂, China, Guangdong, Xinyi City, Dawuling, 1.vi.2002, Cheng Chen leg. (MHBU). Paratypes: 3 ♀♀, same locality as holotype, 31.v.2002, Zhong-Ping Deng leg.; 1.vi.2002, Cui-Feng Li leg.; 6.vii.1988, Feng-Long Jia leg. (MHBU); 2 ♀♀, same locality as holotype, 31.v.2002, Bing-Lan Zhang leg.; 7.vii.1988, He-Xiang Zhou leg. (SYSU).

##### Diagnosis.

This new species is similar to *Stenochinus cylindricus*, but can be distinguished from the latter by antennomeres VII–XI wider than long, pronotum moderately projecting anteriorly (Fig. 5), shape of male genitalia different (Figs 13–16) (in *Stenochinus cylindricus*, antennomeres VIII–XI wider than long (Fig. 10), pronotum strongly projecting anteriorly (Fig. 6)).

##### Etymology.

The specific name is derived from the type locality of this species.

##### Description.

Male (Fig. 18). Body length 11.0 mm, elongate, subcylindrical. Head, elytra, legs reddish brown, pronotum brown, antennae and mouthparts yellowish brown; scale-like hairs surface pale golden. Head transversely subelliptical, surface densely punctate; clypeus transverse, weakly convex in middle, clypeogenal suture grooved, frontoclypeal suture invisible; genae weakly raised; frons wide, distance between eyes 2.53 times as wide as the transverse diameter of an eye in dorsal view. Eyes medium-sized, weakly protruding, with groove along inner and posterior margins. Antennae (Fig. 8) clavate, antennomeres VII–XI wider than long, antennomeres XI oval, length ratio of antennomeres II–XI as 0.16 : 0.17 : 0.12 : 0.10 : 0.09 : 0.09 : 0.10 : 0.13 : 0.10 : 0.23. Maxillary palpomere IV moderately expanded. Pronotum (Fig. 2) 1.14 times as long as wide, widest shortly before middle; anterior margin weakly reflexed; posterior margin weakly bisinuate, with deep emargination in middle; both sides steeply inclined downward, lateral margins sinuate before posterior angles; anterior angles acute and directed anteriorly, posterior angles obtuse and directed posteriorly; disc subelliptically projected in anterior parts, projecting area weakly impressed, surface roughly and deeply punctuate, punctures often fused with another. Scutellum subquadrate, glabrous. Elytra 2.51 times as long as wide, widest at apical 1/3, 2.80 times as long as and 1.11 times as wide as pronotum; dorsum convex but flattened in lateral view; disc with rows of large, deep and subquadrate punctures, each puncture with a granule on each lateral margin; intervals somewhat transversely wrinkled, scale-like hairs shorter and narrower than those on pronotum. Ventral side covered with dense punctures and scale-like hairs, which shorter than those on pronotum. Legs relatively short, length ratio of metatarsomeres I–IV as 0.40 : 0.33 : 0.21 : 0.67. Male genitalia (Figs 13–14) strongly curved in middle in lateral view, 2.29 mm long, 0.43 mm wide; apicale 0.77 mm long, weakly curved in lateral view.

Female (Fig. 19): Body 11.0–12.5 mm, dark brown. Antennae (Fig. 9) thicker, length ratio of antennomeres II–XI as 0.12 : 0.14 : 0.11 : 0.10 : 0.07 : 0.08 : 0.09 : 0.12 : 0.11 : 0.26. Pronotum (Fig. 3) 1.10 times as long as wide. Elytra 2.42 times as long as wide.

#### Stenochinus cylindricus

Taxon classificationAnimaliaColeopteraTenebrionidae

(Gebien, 1914)

Figs 4 , 6 , 10 , 15–16 , 20

Dicraeosis cylindricus Gebien, 1914: 9.

##### Material examined.

2 ♂♂, 5 unsexed, China, Taiwan, Hualien county, Xiulin township, Tailuge, 60–500 m, 13.v.2010, Wen-Yi Zhou leg. (MHBU); 1 unsexed, China, Taiwan, Taitung city, Lianren town, Shouka, 450 m, 16.vi.2010, Wen-Yi Zhou leg. (MHBU).

##### Distribution.

South China: Taiwan.

#### Stenochinus leprosus

Taxon classificationAnimaliaColeopteraTenebrionidae

(Ren & Yang, 2004)

Dicraeosis leprosus Ren & Yang, 2004: 317.

##### Type material examined.

Holotype: ♂, China, Guangxi, Fangcheng, Fulong, 500 m, 24.v.1999, Da-Jun Liu leg. (IZCAS).

##### Distribution.

South China: Guangxi.

## Supplementary Material

XML Treatment for Stenochinus apiciconcavus

XML Treatment for Stenochinus xinyicus

XML Treatment for Stenochinus cylindricus

XML Treatment for Stenochinus leprosus
